# Evaluation of the Safety and Protection Efficacy of *spiC* and *nmpC* or *rfaL* Deletion Mutants of *Salmonella* Enteritidis as Live Vaccine Candidates for Poultry Non-Typhoidal Salmonellosis

**DOI:** 10.3390/vaccines7040202

**Published:** 2019-11-30

**Authors:** Qiuchun Li, Yue Zhu, Jingwei Ren, Zhuang Qiao, Chao Yin, Honghong Xian, Yu Yuan, Shizhong Geng, Xinan Jiao

**Affiliations:** 1 Key Laboratory of Prevention and Control of Biological Hazard Factors (Animal Origin) for Agri-food Safety and Quality, Ministry of Agriculture of China, Yangzhou University, Yangzhou 225009, China; qcli@yzu.edu.cn (Q.L.); M160371@yzu.edu.cn (Y.Z.); MZ120170979@yzu.edu.cn (J.R.); MX120180689@yzu.edu.cn (Z.Q.); dx120180114@yzu.edu.cn (C.Y.); MZ120181147@yzu.edu.cn (H.X.); MX120170779@yzu.edu.cn (Y.Y.); gszhong@yzu.edu.cn (S.G.); 2 Jiangsu Key Lab of Zoonosis/Jiangsu Co-Innovation Center for Prevention and Control of Important Animal Infectious Diseases and Zoonoses, Yangzhou University, Yangzhou 225009, China; 3 Joint International Research Laboratory of Agriculture and Agri-Product Safety, Yangzhou University, Yangzhou 225009, China

**Keywords:** *Salmonella enterica* serovar Enteritidis (*S.* Enteritidis), *spiC*, *nmpC*, *rfaL*, live attenuated vaccine

## Abstract

*Salmonella enterica* serovar Enteritidis (*S.* Enteritidis) is a host-ranged pathogen that can infect both animals and humans. Poultry and poultry products are the main carriers of *S.* Enteritidis, which can be transmitted to humans through the food chain. To eradicate the prevalence of *S.* Enteritidis in poultry farms, it is necessary to develop novel vaccines against the pathogen. In this study, we constructed two vaccine candidates, CZ14-1*∆spiC∆nmpC* and CZ14-1*∆spiC∆rfaL*, and evaluated their protective efficacy. Both mutant strains were much less virulent than the parental strain, as determined by the 50% lethal dose (LD_50_) for three-day-old specific-pathogen free (SPF) White Leghorns and Hyline White chickens. Immunization with the mutant candidates induced highly specific humoral immune responses and expression of cytokines IFN-γ, IL-1β, and IL-6. In addition, the mutant strains were found to be persistent for almost three weeks post-infection. The survival percentages of chickens immunized with CZ14-1*∆spiC∆nmpC* and CZ14-1*∆spiC∆rfaL* reached 80% and 75%, respectively, after challenge with the parental strain. Overall, these results demonstrate that the two mutant strains can be developed as live attenuated vaccines.

## 1. Introduction

The *Salmonella enterica* serovar Enteritidis (*S.* Enteritidis) is one of the most common *Salmonella* serotypes causing human infections worldwide [1,2]. Human salmonellosis is frequently caused by consumption of *Salmonella*-contaminated foods. From 2015 to 2017, the proportion of human salmonellosis caused by *S.* Enteritidis increased from 54.7% to 61.2% in the EU (EFSA, 2018). In the USA, *S*. Enteritidis was the predominant serotype in reported human *Salmonella* infections from 2011 to 2016, and it was also the most common serotype in clinical chicken isolates [1]. In China, our surveillance results over the last 10 years showed that about 30% of *Salmonella* isolates from poultry meat were *S*. Enteritidis. In addition, epidemiological reports implicated that human *S.* Enteritidis cases are frequently caused by the consumption of contaminated chicken products [3].

Since poultry and poultry products, including eggs, have been identified as the major source of *S.* Enteritidis in human foods worldwide, there is an urgent need for efficient programs to control *Salmonella* infections in poultry. Strict biosecurity measures, including tight control of the housing environment, feed quality, water supply, management pattern, and complete exclusion of sources of infection (rodents, insects, wild birds, etc.), should be applied to prevent the introduction of the pathogen into poultry farms [4,5]. However, the high costs of these measures restrict their application in many developing countries. Antimicrobial therapy is another strategy that can be used to control *Salmonella* infection, but the excessive use of antibiotics has led to the emergence of multidrug-resistant strains [6,7]. Bacteriophage therapy is another method developed to control *Salmonella* infection in poultry by reducing the bacterial load in chickens, but treatment of bacteriophage can cause phage-resistant *S*. Enteritidis [8,9]. Therefore, vaccination is an efficient strategy for the control of *Salmonella* infections in poultry. To date, some *S.* Enteritidis vaccines have been developed and used in the poultry industry. Application of these vaccines decreased the incidence of *S*. Enteritidis in poultry flocks [10]. However, administration of live attenuated vaccines is not yet able to prevent the spread of *S.* Enteritidis in poultry flocks in China [11]. New vaccines are needed to prevent the infection and the spread of *S*. Enteritidis in the poultry industry.

SpiC is an SPI-2-encoded effector protein that is secreted by SPI-2 type III secretion system (T3SS) of *Salmonella* into macrophages [12]. Deletion of *spiC* has been confirmed to significantly reduce *Salmonella* virulence in mice and chickens [12,13,14]. Furthermore, the *spiC*-deleted *S.* Pullorum strain has been evaluated as a potential vaccine candidate in chickens [13,15]. To distinguish vaccinated animals from naturally infected ones, the so-called DIVA (differentiating infected from vaccinated animals) strategy has been successfully applied in vaccine development [16]. Vaccine candidates with the genes encoding the outer segment of bacterial lipopolysaccharide (LPS) and outer membrane proteins deleted can be developed as DIVA vaccines, which can be differentiated from natural infection by using available diagnostic serological tests [17,18]. The *rfaL* gene encodes a membrane enzyme involved in the synthesis of LPS, and deletion of the gene changes smooth LPS to rough LPS, which cannot react with the antibodies against the O antigen [15,19]. In addition, the *rfaL*-deleted mutant of *S.* Typhimurium was also attenuated in mice [20]. OmpD is the most abundant porin in the outer membrane of *Salmonella* involved in inducing the B1b cell antibody response in mice [21,22]. Deletion of the *ompD* gene (also named *nmpC*) slightly reduced the virulence of *S.* Typhimurium in mice. Therefore, both *rfaL* and *nmpC* can be chosen as target genes for construction of DIVA vaccine candidates based on deletion of *spiC* in *S.* Enteritidis.

In the present study, we constructed two *S.* Enteritidis vaccine candidates and evaluated their protective efficacy in chickens. The mutants CZ14-1*∆spiC∆nmpC* and CZ14-1*∆spiC∆rfaL* were generated by λ-Red-mediated recombination [23] and homologous recombination using suicide vector pGMB151 [24]. The feasibility of these two mutants as live attenuated vaccines was subsequently evaluated by analyzing virulence, immune response, protective efficacy, etc.

## 2. Materials and Methods

### 2.1. Bacterial Strains and Development of S. Enteritidis Mutant Strains

*S.* Enteritidis CZ14-1 is a clinical strain that was obtained from chicken eggs from a poultry farm in the Jiangsu Province of China in 2016 [11]. It is a wild type virulent strain with resistance to Nalidixic acid. Deletion of *spiC* in CZ14-1 was performed using the suicide vector pGMB151 based on homologous recombination, as previously described [24]. Either *rfaL* or *nmpC* was subsequently deleted using the one-step activation method of the λ-Red mutation system [22]. The open reading frame (ORF) of the targeted genes were completely deleted, and this was confirmed by PCR analysis and sequencing. All of the strains were cultured in Luria-Bertani (LB) agar medium, LB broth, and Brilliant green agar (Becton Dickinson) at 37 °C.

### 2.2. Chickens

The specific-pathogen free (SPF) White Leghorns and Hyline White chickens were purchased from Jinan Sipaffrey Poultry Co., Ltd. (Jinan, Shangdong, China). The 3-day old chickens were confirmed as free from *Salmonella* infection by both bacteriological examination and serum detection. Each group of chickens was housed in separate rearing isolators and supplied with commercial feed and drinking water. The food and the water for chickens were tested to be *Salmonella* negative. All of the animal experiments and management procedures were undertaken with the permission of the Animal Welfare and Ethics Committees of Yangzhou University (Approval No. SYXK(Su)2016-0020) and complied with the guidelines of the institutional administrative committee and the ethics committee for laboratory animals.

### 2.3. Virulence Assessment

To evaluate the virulence of the two mutants (CZ14-1*∆spiC∆nmpC* and CZ14-1*∆spiC∆rfaL*) in chickens, the 50% lethal doses (LD_50_) were determined using 50 chickens for each strain randomly assigned into five groups. Each group was inoculated intramuscularly with 10-fold dilutions of the mutants from 1 × 10^5^ to 1 × 10^9^ CFU. Another group of 50 was inoculated intramuscularly with the parental strain CZ14-1 from 1 × 10^4^ to 1 × 10^8^ CFU. In addition, ten chickens were inoculated with 200 µL of phosphate-buffered saline (PBS) via the same route. Birds that died were recorded daily for three weeks post-infection, and the LD_50_ was calculated using the Reed–Muench method [25].

### 2.4. Biochemical Test and Growth Ability In Vitro

To investigate the effect of deleted genes on the biological properties of *S.* Enteritidis mutants, a biochemical test was performed using an API20E plate (BioMérieux, France), and the results compared to those for the parental strain CZ14-1. In addition, the growth ability was determined by measuring the optical density (OD_600_) of each strain cultured in 15 mL of LB broth at 37 °C with shaking at 180 rpm. The OD_600_ was monitored every hour for 18 h as previously described [26].

### 2.5. Bacterial Vaccination and Changes in Body Weight in Chickens

A total of 60 7-day old Hyline White chickens were randomly divided into three groups (*n* = 20 for each group): CZ14-1*∆spiC∆nmpC*-vaccinated group, CZ14-1*∆spiC∆rfaL*-vaccinated group, and non-vaccinated control group. The vaccinated groups were immunized intramuscularly with 1 × 10^5^ CFU of one of the mutant strains in 100 µL PBS, while the control group was only inoculated with PBS. At 14 days old, the chickens were again immunized via the same route and the same dose of bacteria. The body weights of these chickens were then measured at 7, 14, and 21 days after the second vaccination.

### 2.6. Persistence and Clearance of Bacteria in Chicken Tissues and Organs

A total of 90 3-day old Hyline White chickens were randomly assigned into three groups (*n* = 30 for each group). Each group was immunized intramuscularly with 1 × 10^5^ CFU of CZ14-1, CZ14-1*∆spiC∆nmpC*, or CZ14-1*∆spiC∆rfaL*. Liver, spleen, ileum, and cecum samples from five chickens from each group were aseptically collected at 1, 3, 7, 14, and 21 days post immunization. The samples were weighed and suspended in 1 mL of PBS for homogenization. The homogenates were diluted serially and subsequently inoculated on Brilliant blue agar plates for bacterial recovery at 37 °C. After overnight cultivation, the bacterial number was counted and reported as log_10_ CFU/g.

### 2.7. Immune Response

The humoral immune response was evaluated through measurement of IgG titers specifically against *S.* Enteritidis by indirect enzyme-linked immunosorbent assay (ELISA) using the heat-killed CZ14-1 strain as the coating antigen. Serum samples were collected at 7, 14, and 21 days post immunization and then serially diluted to be considered as the primary antibody. Horseradish peroxidase (HRP)-conjugated goat anti-chicken IgG (1:10,000 dilution, Sigma-Aldrich) was used as the secondary antibody. The HRP activity was determined using 3,3′,5,5′-tetramethylbenzidine (TMB, Sigma-Aldrich), and the OD_450_ value was determined with an ELISA reader (BioTek, USA).

To evaluate the T-cell immune response and inflammatory response induced by the vaccine strains, the expression of cytokines IFN-γ, IL-1β, IL-6, and CXCLi1 was measured by qRT-PCR analysis with Taqman^TM^ probes as previously described [27]. Total RNA was extracted from spleen samples collected at 7, 14, and 21 days post-vaccination using an RNA plus Mini kit (Qiagen, Germany) according to the manufacturer’s instructions. The RNA was reverse transcribed into cDNA and subjected to qRT-PCR analysis, which was performed in triplicate and repeated three times.

### 2.8. Immune Protection Assessment

To evaluate the protective efficacy of the two vaccine candidates, 60 7-day old Hyline White chickens were randomly divided into three groups of 20 chickens (groups A, B, and C). Groups A and B were immunized intramuscularly with 1 × 10^5^ CFU of the mutants CZ14-1*∆spiC∆nmpC* and CZ14-1*∆spiC∆rfaL*, respectively. Group C received 100 µL of PBS as the negative control. One week post-infection, groups A and B were immunized again with the same mutants using the same route and dose. After one week, all of the groups were challenged with 2 × 10^9^ CFU of CZ14-1. The number of surviving chickens was recorded, and clinical symptoms were observed daily for three weeks. In addition, to examine the persistence and the clearance of the wild-type strain in the vaccinated chickens, 90 7-day old chickens were divided into three groups of 30 chickens and subjected to the same immunization and challenge process as described above. After challenging, five chickens from each group were killed at 3, 7, 14, 21, 28, and 35 days post-challenge, liver, spleen, ileum, and cecum samples were collected, and the bacterial number was counted on Brilliant green agar plates, as mentioned above.

### 2.9. Statistical Analysis

All data are described as mean ± standard error of the mean (SEM) unless otherwise specified and were analyzed using GraphPad Prism version 7.0. A *p* value of less than 0.05 was considered statistically significant.

## 3. Results

### 3.1. Virulence of the Two Vaccine Candidates

The virulence of the parental and the two mutant strains was evaluated in three-day-old SPF White Leghorns and Hyline White chickens. As shown in Table 1, the LD_50_ of the CZ14-1*∆spiC∆nmpC* (1.58 × 10^7^ CFU) and CZ14-1*∆spiC∆rfaL* (7.76 × 10^6^ CFU) strains were 710-fold and 346-fold higher than that of parental strain CZ14-1 (2.24 × 10^4^ CFU) in White Leghorn chickens; in Hyline White chickens, the LD_50_ of the CZ14-1*∆spiC∆nmpC* (2.57 × 10^7^ CFU) and CZ14-1*∆spiC∆rfaL* (4.07 × 10^6^ CFU) strains were 2030-fold and 407-fold higher than that of the CZ14-1 strain (1.26×10^4^ CFU) Table 2. The results indicate that both mutant strains were attenuated and that CZ14-1*∆spiC∆nmpC* was highly attenuated compared to CZ14-1*∆spiC∆rfaL*. In addition, the Hyline White chickens were more sensitive to *S*. Enteritidis than the SPF chickens.

### 3.2. Biochemical Property and Growth Ability of Mutant Strains

**Figure 1:**
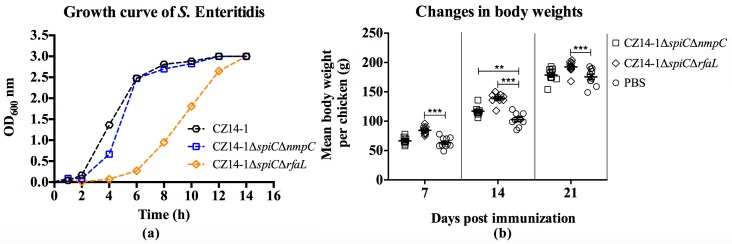
Changes in growth ability of mutants in Luria-Bertani (LB) broth (**a**) and body weight of chickens post immunization (**b**). (**a**) The growth curves of the wild type stain CZ14-1, the mutant strains CZ14-1*∆spiC∆nmpC* and CZ14-1*∆spiC∆rfaL* in LB broth. Deletion of *rfaL* in CZ14-1*∆spiC* caused the decreased growth ability of *S.* Enteritidis in LB broth. (**b**) Changes in mean body weights of chickens after immunization. The vaccinated groups were intramuscularly inoculated with 1 × 10^5^ CFU mutants in seven-day-old chickens, and control group received 100 μL phosphate-buffered saline (PBS). ** *p* ≤ 0.01; *** *p* ≤ 0.001.

The biochemical test performed using the API20E plate reflected that the mutated genes had no significant effect on bacterial metabolism (unpublished). Mutation of *rfaL* in CZ14-1*∆spiC∆rfaL* caused a deficiency in *S.* Enteritidis in utilizing mannitol. Moreover, the bacterial growth data showed that mutation of *rfaL* decreased growth under laboratory conditions. However, the growth curve of CZ14-1*∆spiC∆nmpC* was similar to that of the parental strain, which showed that deletion of *nmpC* did not perturb the bacterial growth in vitro Figure 1a.

### 3.3. Changes in Body Weight Following Immunization

The mean body weights of the chickens in the two vaccinated groups and the PBS group at 7, 14, and 21 days post-vaccination are shown in Figure 1b. Chickens in both vaccinated groups showed increased body weight compared to that of the PBS group. Furthermore, the bodyweight of chickens in the CZ14-1*∆spiC∆rfaL* group was significantly higher than that of the PBS group. No significant difference was detected between the CZ14-1*∆spiC∆nmpC*-vaccinated group and the PBS group at 7 and 21 days post-vaccination.

### 3.4. Bacterial Colonization and Persistence in Chicken Tissues and Organs

**Figure 2:**
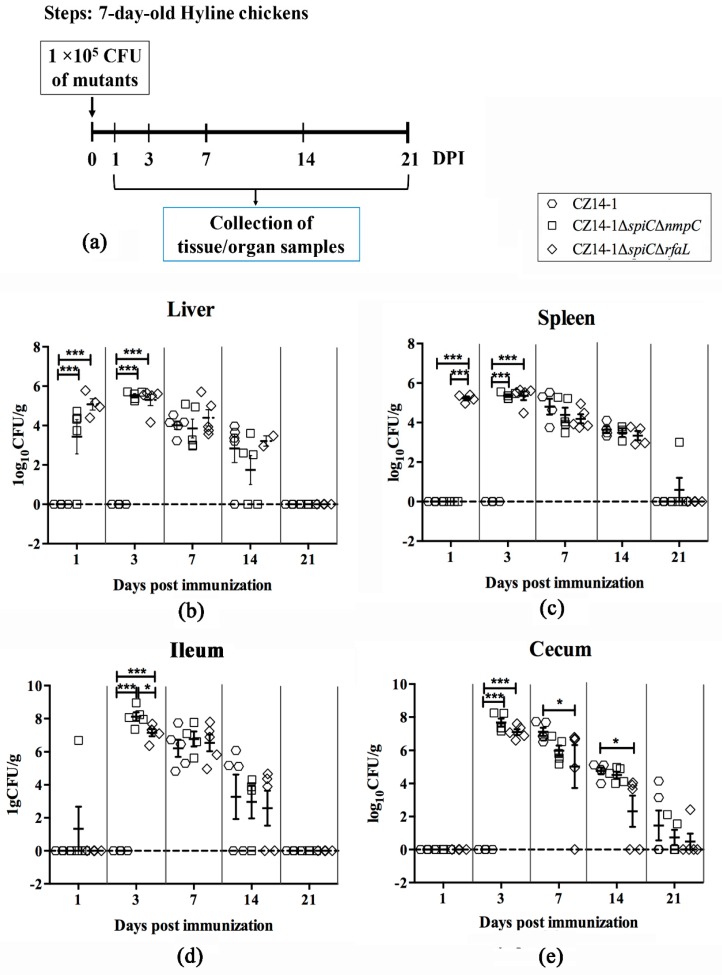
Bacterial colonization in tissues and organs of immunized chickens. Chickens were immunized by parental strain CZ14-1, vaccine candidates CZ14-1*∆spiC∆nmpC*, and CZ14-1*∆spiC∆rfaL*, respectively (**a**). Bacterial colonization in liver (**b**), spleen (**c**), ileum (**d**), and cecum (**e**) of the immunized chickens. The number of bacteria was determined and expressed as log_10_CFU/g. Data are expressed as the mean ± SEM, * *p* ≤ 0.05; *** *p* ≤ 0.001.

All of the tissues and organs collected from chickens inoculated with PBS were negative for bacterial recovery. However, although the virulence of the two mutant strains was significantly decreased compared to the parental strain, the colonization ability of both mutant strains in all four tissue and organ types tested was significantly stronger than that of the wild-type strain at 3 days post-immunization (Figure 2). Bacterial recovery was not detected in tissue and organ types tested from the wild-type-infected group at 1 and 3 days post-immunization. While ≥4/5 (four out of five chickens) of CZ14-1*∆spiC∆nmpC* and all five of the CZ14-1*∆spiC∆rfaL*-immunized chickens were found to be positive for *Salmonella* in the liver at 1 and 3 days post-immunization (Figure 2a), the CZ14-1*∆spiC∆rfaL* exhibited faster invasion into the spleen than the parental strain and CZ14-1*∆spiC∆nmpC* (Figure 2b). At seven and 14 days post-immunization, the bacterial counts in the spleen, the liver, and the ileum were similar among the three groups (Figure 2a–c), but there was a significant difference between cecum from the wild-type-infected group and that from chickens vaccinated with CZ14-1*∆spiC∆rfaL* (Figure 2d). At 21 days post-immunization, bacteria were almost eliminated in the liver and the ileum in all three groups. Only one chicken vaccinated with CZ14-1*∆spiC∆nmpC* was found to be *Salmonella*-positive in the spleen (Figure 2c). However, in the cecum, a low level of bacterial recovery could still be detected in all three groups (Figure 2d).

### 3.5. Immune Response

**Figure 3:**
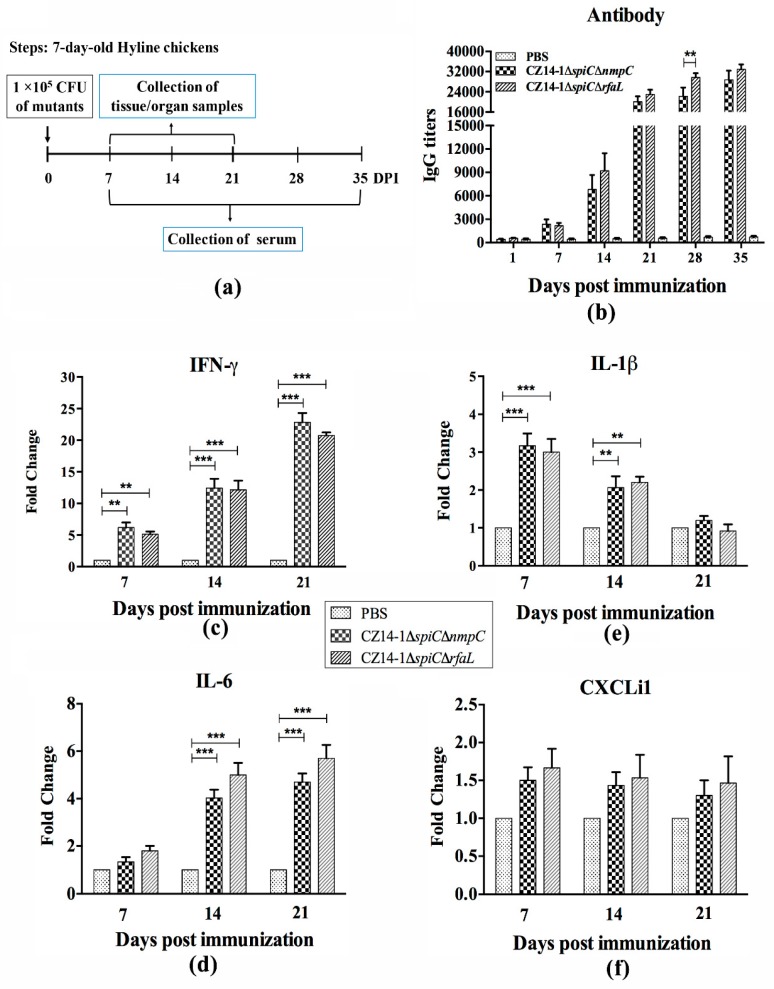
Immune response in spleens from chickens immunized with vaccine candidates. The 7-day-old Hyline White chickens were immunized with 1 × 10^5^ CFU of mutants (**a**), and the antisera were subjected to test IgG titers at 1, 7, 14, 21, 28, and 35 days post immunization (**b**). The total RNA was extracted from spleens of chickens vaccinated by mutants at 7, 14, and 21 days post immunization and subsequently subjected to the qRT-PCR analysis for detection the expression levels of IFN-γ (**c**), IL-6 (**d**), IL-1β (**e**), and CXCLi1 (**f**), respectively. ** *p* ≤ 0.01; *** *p* ≤ 0.001.

To evaluate the humoral immune response induced by the two vaccine candidates, serum IgG levels were determined using an indirect ELISA assay. In both of the vaccinated groups, the serum IgG against *S.* Enteritidis was detected at 7 days post immunization and increased dramatically at 14 and 21 days post immunization then remained stable from 21 to 35 days post immunization (Figure 3a, 3b). At 28 days post immunization, the serum IgG level was significantly higher in the CZ14-1*∆spiC∆rfaL*-immunized group than that in the CZ14-1*∆spiC∆nmpC*-immunized chickens (Figure 3b).

qRT-PCR analysis of cytokine expression levels in the spleen showed that both vaccinated groups displayed higher IFN-γ levels than the control group at 7, 14, and 21 days post-immunization, and no difference was detected between the two vaccinated groups (Figure 3c). The expression of IL-1β was higher in the two vaccinated groups than the PBS group at 7 and 14 days post immunization (Figure 3e), while IL-6 showed higher expression at 14 and 21 days post-immunization in the vaccinated groups than that in the PBS group (Figure 3d). Immunization with the two mutant strains had no effect on the expression of CXCLi1, which was similar in the vaccinated groups and the PBS group (Figure 3f).

### 3.6. Immune Protection

The survival percentages for the chickens vaccinated intramuscularly with CZ14-1*∆spiC∆rfaL* and CZ14-1*∆spiC∆nmpC* followed by challenge with the wild-type strain CZ14-1 are shown in Table 3. Four and five chickens died in groups A and B, respectively, following the challenge with the parental strain. However, 19 out of 20 non-vaccinated chickens died in group C. The clinical symptoms in the vaccinated group were slight and temporary after the challenge compared with the severe diarrhea seen in the non-vaccinated chickens.

**Figure 4:**
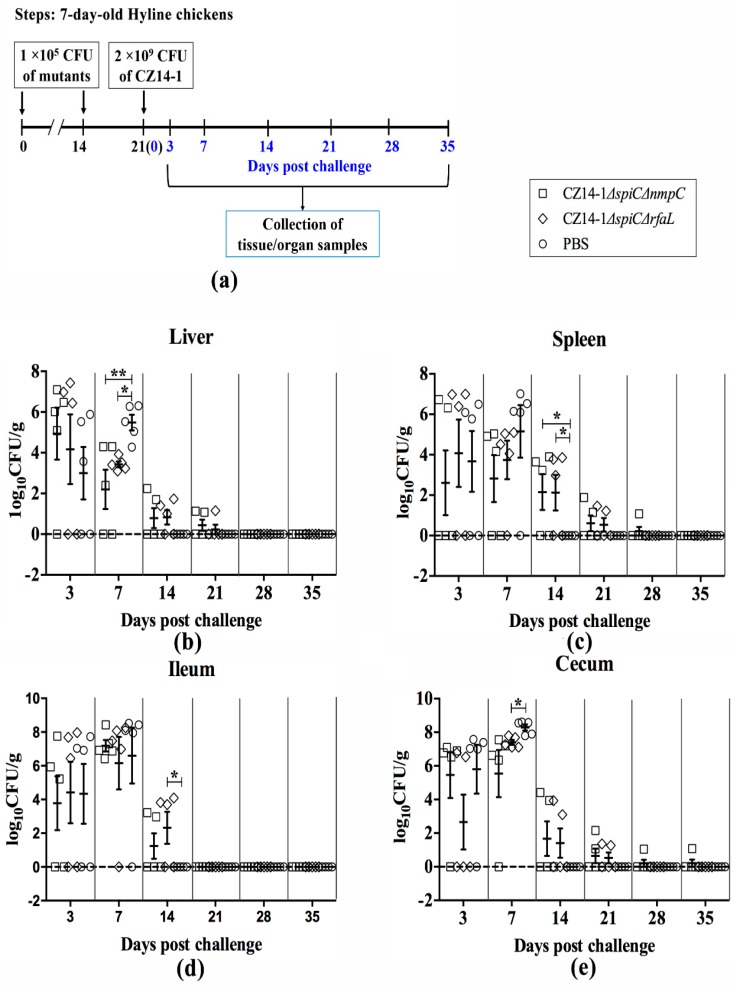
Bacterial colonization in challenged chickens. The chickens were immunized with vaccine candidates CZ14-1*∆spiC∆nmpC* and CZ14-1*∆spiC∆rfaL*, respectively, and then challenged with 2 ×10^9^ CFU of CZ14-1 (**a**). Bacterial colonization in liver (**b**), spleen (**c**), ileum (**d**), and cecum (**e**) of vaccinated chickens after challenge with parental strain CZ14-1. The number of bacteria was determined and is represented as log_10_ CFU/g. Data are expressed as the mean ± SEM, * *p* ≤ 0.05; ** *p* ≤ 0.01.

To detect the bacteria eradication ability of the vaccinated groups, liver, spleen, ileum, and cecum were collected for bacterial recovery and counting. As shown in Figure 4, the bacterial number was high at 3 and 7 days post-challenge but decreased dramatically at 14 days post-challenge. The bacteria were eradicated at 21, 28, and 35 days post-challenge in the ileum, the liver, and the spleen, respectively (Figure 4b–d). In the cecum, only one chicken in the CZ14-1*∆spiC∆nmpC*-vaccinated group had almost 10 CFU/g of *Salmonella* at 35 days post-challenge (Figure 4e). Since most of the chickens in the non-vaccinated group were dead at 7 days post-challenge, no data were collected for the following days in this group.

## 4. Discussion

Until now, three major types of vaccines, namely, killed bacteria, subunit vaccines, and live attenuated vaccines, have been developed to control *S.* Enteritidis in the poultry industry [28,29,30,31,32]. The killed bacteria and subunit vaccines can induce strong antibodies to eradicate extracellular bacteria, but it is difficult for these antibodies to kill the intracellular *S.* Enteritidis, which could be achieved with the live attenuated strains of *Salmonella* [28,32,33]. A live attenuated *Salmonella* vaccine should be attenuated, immunogenic, protective, and allow differentiation of vaccinated chickens from naturally infected flocks [34,35]. In this study, we constructed two mutants with virulence factor (SPI-2 effector) *spiC* mutated combined with a gene involved in the synthesis of LPS (*rfaL*) or out-membrane protein (*nmpC*). Both mutants are highly attenuated, strongly immunogenic, and provide excellent protective efficacy against parental strain attack. In addition, ELISA or plate agglutination test (PAT) can be used to differentiate vaccinated from naturally infected chickens.

A live attenuated vaccine should be avirulent. SpiC is a SPI2-encoded effector involved in the survival of *Salmonella* in macrophages [36,37]. Deletion of *spiC* in *S.* Typhimurium and *S.* Pullorum caused significantly decreased virulence in mice and chickens, respectively [13,37]. Although deletion of *spiC* has been achieved in *S.* Enteritidis C50041, which was confirmed to be less virulent than the parental strain, a mouse-derived isolate [38], in this study, we constructed an *spiC*-deleted strain combined with *rfaL* or *nmpC* gene using chicken-borne isolate CZ14-1. Both double-gene mutants showed greatly decreased virulence compared to that of the parental strain. Furthermore, immunization with the mutants did not affect the chickens’ body weights, leading to better growth than in the non-vaccinated group. No indications of negative effects on the health of the immunized birds were observed.

To eradicate intracellular *Salmonella*, it is critical to stimulate cellular immune responses in the immunized animals [39,40]. At the acute infection stage, the *Salmonella*-infected host can produce both Th1 and Th2 immune responses to diminish the growth of the invading bacteria, but in the second stage of persistent infection, the reduced Th1 response disrupts the balance between Th1 and Th2, and a new equilibrium between the host and persistent *Salmonella* infection is developed and continues [41]. Both mutants in this study can induce increased IFN-γ and IL-6 secretion from 7 to 21 days post-immunization, indicating that a prolonged Th1 immune response can be induced by these mutants. Furthermore, the antibody against *S*. Enteritidis was produced at 7 days post-immunization, after which production increased and then remained at a stable concentration to kill the extracellular bacteria. However, bacterial shedding is another key point for live attenuated vaccines [42]. In this study, both mutants were almost eliminated in the liver, the spleen, and the ileum at 21 days post-immunization, and the bacteria were not detected at 28 days post-immunization. These results demonstrate that both mutants can be eliminated from the chickens but induce strong cellular immune and humoral responses.

## 5. Conclusions

Our data demonstrated that the two mutants could significantly protect the chickens from virulent parental strain challenge. The lower virulence and the higher protective efficacy showed that CZ14-1*∆spiC∆nmpC* was better than CZ14-1*∆spiC∆rfaL.* However, for the eradication of the colonized parental strain in tissues, the group immunized with CZ14-1*∆spiC∆rfaL* was better than that immunized with CZ14-1*∆spiC∆nmpC*. Since the *spiC* deletion mutant of *Salmonella* has proven to be an appropriate vaccine candidate against *S.* Pullorum/*S.* Gallinarum infection [13,14], the present two double-mutants show potential for use as vaccines against *S.* Enteritidis infection in poultry.

## Figures and Tables

**Table 1:** The LD50 of *S.* Enteritidis CZ14-1, CZ14-1*∆spiC∆nmpC* and CZ14-1*∆spiC∆rfaL* in three-day-old White Leghorn chickens after intramuscular immunization.

Strains	Challenge Dose (CFU)	No. of Deaths/Total No. of Chickens	LD_50_ (CFU)
CZ14-1	1 × 10^8^	10/10	2.24 × 10^4^
	1 × 10^7^	9/10	
	1 × 10^6^	7/10	
	1 × 10^5^	6/10	
	1 × 10^4^	4/10	
CZ14-1*∆spiC∆nmpC*	1 × 10^10^	10/10	1.58 × 10^7^
	1 × 10^9^	10/10	
	1 × 10^8^	10/10	
	1 × 10^7^	5/10	
	1 × 10^6^	2/10	
CZ14-1*∆spiC∆rfaL*	1 × 10^10^	10/10	7.76 × 10^6^
	1 × 10^9^	10/10	
	1 × 10^8^	10/10	
	1 × 10^7^	7/10	
	1 × 10^6^	3/10	

**Table 2:** The LD50 of *S.* Enteritidis CZ14-1, CZ14-1*∆spiC∆nmpC* and CZ14-1*∆spiC∆rfaL* in three-day-old Hyline White chickens after intramuscular immunization.

Strains	Challenge Dose (CFU)	No. of Deaths/Total No. of Chickens	LD_50_ (CFU)
CZ14-1	1 × 10^8^	10/10	1.26 × 10^4^
	1 × 10^7^	10/10	
	1 × 10^6^	9/10	
	1 × 10^5^	6/10	
	1 × 10^4^	5/10	
CZ14-1*∆spiC∆nmpC*	1 × 10^9^	10/10	2.57 × 10^7^
	1 × 10^8^	10/10	
	1 × 10^7^	5/10	
	1 × 10^6^	2/10	
	1 × 10^5^	0/10	
CZ14-1*∆spiC∆rfaL*	1 × 10^9^	10/10	4.07 × 10^6^
	1 × 10^8^	10/10	
	1 × 10^7^	6/10	
	1 × 10^6^	3/10	
	1 × 10^5^	0/10	

**Table 3:** Protective efficiency of two vaccine candidates in Hyline White chickens.

Group	Vaccination		No.	Challenge			Survivors/Total (Survival Rate)
	Strain	Dose (CFU)		Strain	Route	Dose	
A	CZ14-1*∆spiC∆nmpC*	1 × 10^5^	20	CZ14-1	i.m.^a^	2 × 10^9^	16/20 (80%)
B	CZ14-1*∆spiC∆rfaL*	1 × 10^5^	20	CZ14-1	i.m.	2 × 10^9^	15/20 (75%)
C	PBS	-	20	CZ14-1	i.m.	2 × 10^9^	1/20 (5%)

^a^ represents the intramuscularly inoculation.
